# Impact of Extracorporeal Membrane Oxygenation on Right Ventricular Function After Heart Transplantation

**DOI:** 10.3389/fcvm.2022.938442

**Published:** 2022-07-15

**Authors:** Cheng Zhao, Xing Hao, Chao Xue, Yichen Zhao, Jie Han, Yixin Jia, Xiaotong Hou, Jiangang Wang

**Affiliations:** ^1^Department of Cardiac Surgery, Beijing Anzhen Hospital, Capital Medical University, Beijing, China; ^2^Department of Intensive Care, Beijing Anzhen Hospital, Capital Medical University, Beijing, China; ^3^Department of Ultrasonography, Beijing Anzhen Hospital, Capital Medical University, Beijing, China

**Keywords:** extracorporeal membrane oxygenation, cardiopulmonary bypass, heart transplantation, heart failure, right ventricular function

## Abstract

**Aims:**

Acute right ventricular failure remains a common challenging clinical syndrome in heart transplant (HTx) recipients. While extracorporeal membrane oxygenation (ECMO) is a proven strategy for the treatment of this condition, the outcomes after weaning and during follow up remain understudied. We aimed to evaluate the right-sided heart function in ECMO survivors following HTx.

**Methods:**

Between September 2005 and December 2019, 205 patients with end-stage heart failure who underwent standard orthotopic HTx were enrolled. In total, 68 (33.2%) patients were included in the ECMO group and 137 (66.8%) patients were included in the non-ECMO group.

**Results:**

Of the 68 patients in the ECMO group, 42 (61.8%) were successfully weaned from ECMO. After a median follow-up period of 53 months, there were 25 (59.5%) and 27 (23.7%) deaths in the ECMO and non-ECMO groups (*P* = 0.023), respectively. Systolic pulmonary artery pressure (SPAP) before discharge (*P* = 0.003) was the unique predictor of all-cause mortality during follow up. Meanwhile, patients in the ECMO group with more than moderate SPAP increase before discharge had higher mortality than patients in the non-ECMO group without such increase (*P* = 0.005).

**Conclusions:**

Recipient right-sided heart characteristics were strong predictors of ECMO need after HTx. ECMO patients had high mortality in the perioperative and follow-up periods, and the changes in right ventricular function in ECMO patients may be associated with pulmonary vessel injury before and after HTx.

## Introduction

Heart transplantation (HTx) is the gold-standard treatment for end-stage heart failure (HF) (1). The most recent data from the registry of the International Society of Heart and Lung Transplantation (ISHLT) showed 1- and 5-year survival rates of 84.5 and 72.5%, respectively. ISHLT registry data show that acute right ventricular (RV) failure remains a difficult and common clinical syndrome in transplant recipients and is associated with 50% of all cardiac complications and 19% of all early deaths after HTx (2). Increased pulmonary vascular resistance and ischemia-reperfusion injury of the myocardium are well-known risk factors for acute RV failure in HTx recipients (3). RV failure results in dilation, ischemia, and decreased contractility. Decreased pulmonary blood flow and leftward septal shift subsequently lead to lower left ventricular (LV) filling and low output syndrome (LOS). Despite multiple medical treatments, some patients cannot be weaned from cardiopulmonary bypass (CPB).

Extracorporeal membrane oxygenation (ECMO) is a proven strategy for the treatment of patients with LOS after HTx (4); it helps support organ system function in these patients during critical periods and allows the newly transplanted heart to work under less stress and eventually recover from the combined shock caused by ischemia-reperfusion injury and exposure to a previously unknown preload (5).

Considering short-term outcomes, studies have indicated that 45–80% of patients can be considered “ECMO survivors.” However, the changes in pulmonary artery pressure (PAP) and RV function during follow up in ECMO survivors remain unknown. This study aimed to evaluate RV function in ECMO survivors after HTx during follow up.

## Methods

### Study Population

Between September 2005 and December 2019, 242 patients with end-stage HF underwent standard orthotopic HTx (bi-atrial) at Anzhen Hospital. The indications followed the ISHLT guidelines for HTx. Patients with cardiac surgeries or re-transplantation, heart-lung transplantation or heart-kidney transplantation, and ECMO assistance before HTx were excluded. Sixty-eight of the remaining 205 patients that received ECMO after HTx for LOS were included in the ECMO group, while 137 were included in the non-ECMO group. All patients admitted to Anzhen Hospital provided written consent at hospital admission.

### ECMO Procedures

According to the ISHLT guidelines, mechanical circulatory support should be initiated early if weaning from CPB after HTx fails. The decision to use VA-ECMO was made by the experienced heart transplantation team including a cardiac surgeon and intensivist. Indications for VA-ECMO therapy included difficulty weaning from CPB (44 patients, 64.7%) or postoperative refractory cardiogenic shock despite adequate volumes and high doses of inotropes (24 patients, 35.3%), such as norepinephrine, dobutamine, epinephrine, and milrinone. All procedures were performed by trained ECMO team members. Patients were evaluated daily for hemodynamic improvement and the possibility of weaning from circulatory support. Clinical and echocardiographic variables were serially assessed to determine if myocardial recovery occurred, and ECMO weaning was performed in patients who fulfilled our institutional weaning criteria and passed an ECMO weaning trial consisting of tolerance to decreasing and clamping ECMO flow. The protocol for ECMO used in our center has been previously described (6).

### Echocardiography Assessment

Standard echocardiography was performed using commercially available equipment. Cardiac morphology was assessed using diameter and area measurements in standard 4- and 2-chamber views. The LV ejection fraction was calculated using the biplane Simpson method. Valve regurgitation was evaluated using color Doppler flow imaging and was graded as none, mild, mild to moderate, moderate, moderate to severe, and severe according to current guidelines (7, 8). Systolic PAP (SPAP) was calculated by adding the peak tricuspid regurgitation systolic gradient to the estimated central venous pressure. Further, RV function was quantified by fractional area change (FAC), tricuspid annular peak systolic excursion (TAPSE), Tei index of the RV, and tricuspid annulus systolic velocity (S'). FAC was measured via 2-dimensional planimetry in the 4-chamber view, while TAPSE and S' were evaluated using tissue Doppler imaging. The Tei index was evaluated using pulse Doppler imaging.

### Follow Up

Postoperatively, all patients visited every 30 days in the first 6 months, every 60 days from 7 to 12 months, and every 6 months thereafter. The patients were invited for history-taking, physical examination, and echocardiographic evaluation during each visit. Major clinical events included all-cause deaths and major HTx complications such as rejection, infection, liver/kidney dysfunction, thrombus, hemorrhage.

Conventional triple-drug immunosuppressive therapy was administered to all recipients, including corticosteroids, calcineurin inhibitors, and antiproliferative agents, which were routinely used during follow up and approved by the guidelines.

### Statistics

Baseline variables are represented as the median and interquartile range (IQR) for continuous variables and as percentages for categorical variables. The chi-squared and Kruskal–Wallis tests were used to analyze unadjusted associations between treatment variables and outcomes. Logistic regression was used to identify the risk factors for ECMO. Covariates were included in the multivariate logistic regression when their log-rank *P*-values were <0.2. Logistic regression with inverse probability for treatment weighting (IPTW), was used to identify the risk factors for perioperative all-cause death. Doubly robust estimates were used if imbalance still existed after adjusting for baseline variables, which was also defined as augmented inverse propensity weighting (AIPW). Similar covariate adjustments were also used in the Kaplan–Meier analysis and Cox regression to determine all-cause mortality during follow up. R version 3.5.2 was used for all statistical analyses, with the twang R package.

### Ethics Approval

Ethics approval for this study was obtained from the Research Ethics Committee of Beijing Anzhen Hospital, Capital Medical University, Beijing, China (IRB approval no.: 2021096X, approval date: July 6, 2020).

## Results

### Baseline Characteristics

Table 1 outlines the baseline characteristics of the recipients and donors, surgical data, and the recipients' echocardiographic data before HTx. The ischemic (P = 0.026) and CPB (P = 0.003) times were significantly higher in the ECMO group than in the non-ECMO group. The ECMO group had higher SPAP, left atrial diameter, right atrial diameter, RV diameter, and right ventricular outflow tract (RVOT) diameter in the preoperative echocardiographic evaluation (P < 0.05).

**Table 1:** Baseline characteristics.

	**ECMO group (*n* = 68)**	**Non-ECMO Group (*n* = 137)**	***P***
**Recipients' characteristics at hospital admission**			
Age, year (IQR)	49.0 (38.5–55.0)	49.0 (38.0–56.0)	0.661
BMI, (IQR)	23.5 (21.0–27.5)	23.4 (20.6–25.5)	0.477
Male sex, *n* (%)	58 (85.3)	104 (75.9)	0.120
Hypertension, *n* (%)	4 (5.9)	8 (5.8)	0.990
Diabetes mellitus, *n* (%)	9 (13.2)	26 (19.0)	0.304
Hypercholesterolemia, *n* (%)	5 (7.4)	15 (10.9)	0.414
Liver/kidney failure, *n* (%)	2 (2.9)	2 (2.9)	0.743
DCM, *n* (%)	51 (75.0)	96 (70.1)	0.461
Others*, *n* (%)	17 (25.0)	41 (29.9)	0.461
NYHA class, (IQR)	4 (3,4)	4 (3,4)	0.548
**Recipients' laboratory and echocardiographic characteristics before HTx**			
AST, U/L (IQR)	24.6 (22.4–26.6)	24.4 (21.3–27.4)	0.861
ALT, U/L (IQR)	23.4 (20.4–25.7)	23.1 (20.7–25.6)	0.929
TBIL, μmol/L (IQR)	17.4 (15.2–19.2)	17.2 (15.6–18.8)	0.623
DBIL, μmol/L (IQR)	6.16 ± 1.44	6.41 ± 1.27	0.223
CREA, μmol/L (IQR)	80.5 (66.3–96.8)	86.0 (71.0–94.0)	0.776
BNP, pg/ml (IQR)	1469.3 (523.5–2010.5)	1410.3 (511.5–2291.8)	0.654
EF, % (IQR)	27.0 (20.3–31.5)	27.0 (21.0–33.0)	0.496
SPAP, mmHg (IQR)	49.0 (44.3–58.8)	42.2 (33.0–51.0)	**<0.001**
PV, cm/s (IQR)	75.0 (63.0–87.5)	69.0 (55.0–81.2)	0.097
AV, cm/s (IQR)	102.5 (78.5–128.3)	98.0 (75.5–110.0)	0.186
LA diameter, mm, median (IQR)	50.0 (46.0–53.8)	47.0 (42.0–52.0)	**0.013**
RA diameter, mm (IQR)	54.0 (48.3–54.8)	47.0 (42.8–52.0)	**<0.001**
RV diameter, mm (IQR)	30.0 (25.2–34.6)	26.3 (22.0–30.0)	**<0.001**
RVOT, mm (IQR)	36.0 (32.3–38.8)	32.0 (28.0–35.1)	**<0.001**
TR (≥moderate), *n* (%)	57 (83.8)	99 (72.3)	0.068
**Donors' information**			
Donor's age, y, median (IQR)	31.5 (28.3–31.5)	31.5 (28.0–35.5)	0.503
Donor's weight, kg (IQR)	68.1 (61.5–74.5)	66.5 (60.0–75.0)	0.468
Donors male sex, *n* (%)	62 (91.2)	126 (92.0)	0.846
**Surgical data**			
Ischemic time, min (IQR)	365.3 (312.3–413.5)	328.0 (258.0–372.0)	**0.026**
CPB time, min (IQR)	173.9 (136.3–204.5)	148.0 (131.0–170.5)	**0.003**

*Categorical data are reported as n (%) and continuous data are reported as mean ± SD or median (IQR). P -value: χ2 test with exact method for categorical variables and t -test or Mann-Whitney U test for continuous variables. P < 0.05 indicates significant statistical difference (bold values)*.

*BMI, body mass index; DCM, dilated cardiomyopathy; CPB time, cardiopulmonary bypass; AST, aspartate transaminase; ALT, alanine aminotransferase; TBIL, serum total bilirubin; DBIL, serum direct bilirubin; CREA, serum creatinine; BNP, serum type B natriuretic peptide; EF, ejection fraction; SPAP, systolic pressure of pulmonary artery; PV, pulmonary valvular velocity; AV, aortic valvular velocity; LA, left atrial; RA, right atrial; RV, right ventricle; RVOT, right ventricular outflow tract; TR, tricuspid regurgitation*.

*Others including: VHD, 6 (8.8%) patients in ECMO group and 6 (4.4%) patients in non-ECMO group; CAD, 7 (10.3%) patients in ECMO group and 25 (18.2%) patients in non-ECMO group; CHD, 2 (2.9%) patients in ECMO group and 5 (3.6%) patients in non-ECMO group; RCM, 2 (2.9%) patients in ECMO group and 1 (0.7%) patient in non-ECMO group; Tumor, 4 (2.9%) in non-ECMO group*.

### ECMO Outcomes

ECMO cannulation was femoral (arterial)-femoral (vein) for all patients. Of the 68 patients in the ECMO group, 42 (61.8%) were successfully weaned from ECMO (median duration, 4.7 days [IQR 3–6]), and 26 (38.2%) died while on ECMO support. Forty (95.2%) patients in the ECMO group who were successfully weaned survived the perioperative period and were discharged from the hospital.

**Figure 1:**
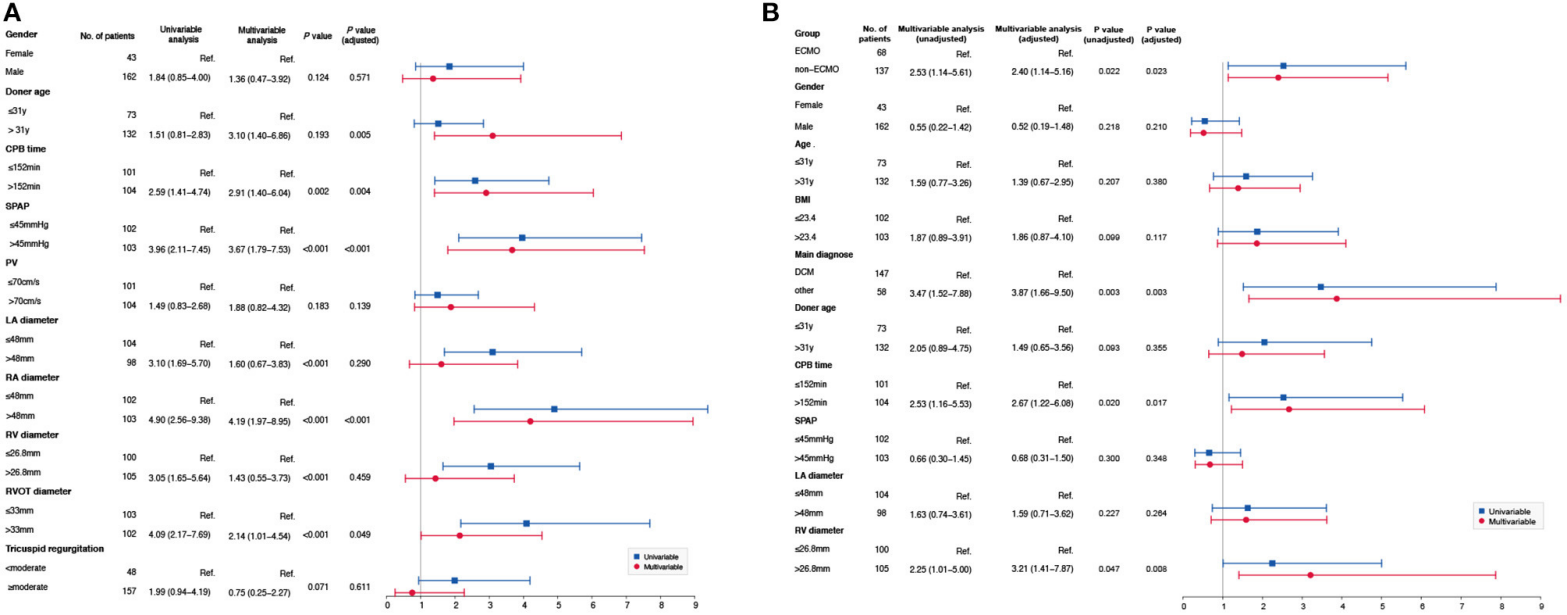
**(A)** Risk factors for ECMO. Multivariate logistic regression analysis showed that donor age (OR 3.10, 95%CI 1.40–6.86, P = 0.005), CPB time (OR 2.91, 95%CI 1.40–6.04, P = 0.004), SPAP (OR 3.67, 95%CI 1.79–7.53, P < 0.001), RA diameter (OR 4.19, 95%CI 1.97–8.95, P < 0.001), and RVOT diameter (OR 2.13, 95%CI 1.01–4.54, P = 0.049) were risk factors for ECMO use. **(B)** Risk factors for perioperative death. Based on the baseline characteristics, multivariate logistic regression analysis showed that ECMO group (OR 2.53, 95%CI 1.14–5.61, P = 0.022), dilated cardiomyopathy (OR 3.47, 95%CI 1.52–7.88, P = 0.003), CPB time (OR 2.53, 95%CI 1.16–5.53, P = 0.020) and RV diameter (OR 2.25 95%CI 1.01–5.00, P = 0.047) were risk factors for perioperative death before AIPW. However, dilated cardiomyopathy (OR 3.87, 95%CI 1.66–9.50, P = 0.003), CPB time (OR 2.67, 95%CI 1.22–6.08, P = 0.017), and RV diameter (OR 3.21, 95%CI 1.41–7.87, P = 0.008) were risk factors for perioperative death after AIPW.

Multivariate logistic regression analysis showed that donor age (odds ratio [OR] 3.100, 95% confidence interval [CI] 1.40–6.86, P = 0.005), CPB time (OR 2.912, 95% CI 1.40–6.04, P = 0.004), SPAP (OR 3.672, 95% CI 1.79–7.53, P < 0.001), RA diameter (OR 4.196, 95% CI 1.97–8.95, P < 0.001), and RVOT diameter (OR 2.135, 95% CI 1.01–4.54, P = 0.049) were risk factors for ECMO use (Figure 1A).

### Perioperative Death and Complications

Of the 205 patients, 51 (24.9%) died during the perioperative period, including 28 (41.2%) and 23 (16.8%) in the ECMO and non-ECMO groups, respectively. Table 2 details the perioperative deaths and complications.

**Table 2:** Perioperative deaths and complications.

	**ECMO**	**Non-ECMO**
	**(n = 68)**	**(n = 137)**
**Perioperative death**, **n** **(%)**	**26 (38.2 of total)**	**23 (16.8 of total)**
Rejection, n (%)	13 (50.0 of death)	13 (56.5 of death)
Infection, n (%)	3 (11.5 of death)	2 (8.7 of death)
Liver/Kidney failure, n (%)	10 (38.5 of death)	1 (4.3 of death)
Other, n (%)	1 (3.8 of death)	7 (30.4 of death)
**Perioperative complications**
Hemorrhage, n (%)	28 (41.2 of total)	5 (3.6 of total)
Embolism, n (%)	7 (10.3 of total)	1 (0.7 of total)
Infection, n (%)	10 (14.7 of total)	2 (1.5 of total)
Liver/Kidney failure, n (%)	22 (32.4 of total)	7 (5.1 of total)
Neurological, n (%)	5 (7.4 of total)	3 (2.2 of total)

*Other perioperative death reasons contained gastrointestinal hemorrhage, cerebral hemorrhage, cerebral infarction, pulmonary embolism, and unexplained sudden death*.

The ECMO group had a higher mortality rate (*P* < 0.001, 95% CI 0.18–0.45) and a higher proportion of deaths due to liver or kidney dysfunction (*P* = 0.003, 95% CI 0.12–0.56), while there was no significant difference in other causes of death between the groups.

For the overall population, multivariate logistic regression analysis showed that ECMO (OR 2.400, 95% CI 1.14–5.16, *P* = 0.023), CPB time (OR 2.670, 95% CI 1.22–6.08, *P* = 0.017), and RV diameter (OR 3.210, 95% CI 1.41–7.87, *P* = 0.008) were risk factors for perioperative death after adjustment (Figure 1B).

The incidence of rejection (13 patients, 50.0%) and liver/kidney dysfunction (10 patients, 38.5%) was higher in the perioperative period in the ECMO group; a higher incidence of rejection (13 patients, 56.5%) was also observed in the non-ECMO group. A higher incidence of postoperative hemorrhage (*P* < 0.001), embolism (*P* = 0.014), infection (*P* = 0.004), and liver/kidney dysfunction (*P* < 0.001) in the perioperative period was observed in the ECMO group (Table 2).

### Follow Up

After a median follow up of 53 months [IQR 24–105], 25 (59.5%) and 27 (23.7%) deaths occurred in the ECMO and non-ECMO groups, respectively. One year after discharge, 142 (91.0%, 95% CI 0.86–0.95) patients survived, comprising 37 (88.1%, 95% CI 0.79–0.99) patients in the ECMO group and 105 (92.1%, 95% CI 0.82–0.97) patients in the non-ECMO group.

**Figure 2:**
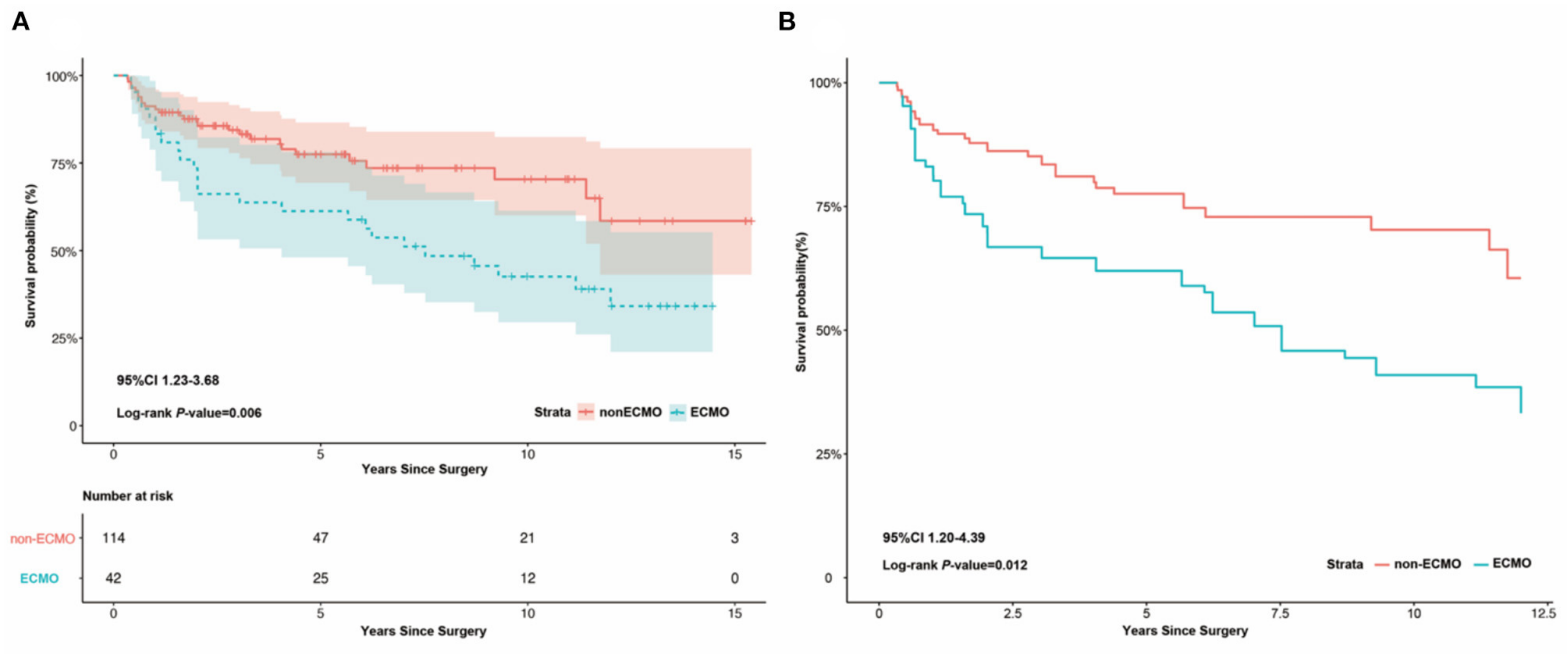
Kaplan-Meier survival analysis between ECMO and non-ECMO group after discharge. **(A)** Kaplan-Meier survival analysis between ECMO and non-ECMO group after discharge, showing the ECMO group had a higher mortality than the non-ECMO group before adjust (log-rank *P* = 0.006, 95%CI 1.23–3.68). **(B)** Kaplan-Meier survival analysis between ECMO and non-ECMO group after discharge, showing the ECMO group had a higher mortality than the non-ECMO group after adjust (log-rank *P* = 0.012, 95%CI 1.20–4.39).

Five years after discharge, 119 (76.3%, 95% CI 0.66–0.81) patients survived, comprising 26 (65.0%, 95% CI 0.79–0.99) patients in the ECMO group and 93 (81.6%, 95% CI 0.82–0.97) in the non-ECMO group. The ECMO group had higher mortality before (OR 2.123, 95% CI 1.23–3.68, *P* = 0.006) and after (OR 2.295, 95% CI 1.20–4.39, *P* = 0.012) adjustment (Figure 2).

Rejection (38.5%), liver/kidney failure (25.0%), and infection (21.2%) were the main causes of death during follow up. Details of the complications during follow up were shown in Table 3.

**Table 3:** Complications during follow-up.

	**Total**	**ECMO**	**non-ECMO**	***P***
	**(*n* = 89)**	**(*n* = 15)**	**(*n* = 74)**	
Infection, *n* (%)	25 (28.1)	5 (33.3)	20 (27.0)	0.625
Fungal, *n* (%)	6 (6.7)	2 (13.3)	4 (5.4)	0.265^*^
Bacterial, *n* (%)	8 (9.0)	3 (20.0)	5 (6.8)	0.254
Viral, *n* (%)	7 (7.9)	0 (0)	7 (9.5)	0.475
Cerebrovascular diseases, *n* (%)	6 (6.7)	0 (0)	6 (8.1)	**0.013**
Thrombus, *n* (%)	6 (6.7)	3 (20.0)	3 (4.1)	0.165
Hemorrhage, *n* (%)	2 (2.2)	0 (0)	2 (2.7)	0.525^*^
Hepatic failure, *n* (%)	2 (2.2)	1 (6.7)	1 (1.4)	0.447^*^
Renal failure, *n* (%)	5 (5.6)	1 (6.7)	4 (5.4)	0.849
CKD, *n* (%)	19 (21.3)	1 (6.7)	18 (24.3)	**0.042**
CAV, *n* (%)	1 (1.1)	1 (6.7)	0 (0)	0.334^*^
HBP after HTx, *n* (%)	33 (37.1)	4 (26.7)	29 (39.2)	0.351
DM after HTx, *n* (%)	21 (23.6)	4 (26.7)	17 (23.0)	0.762
Tumor, *n* (%)	1 (1.1)	1 (6.7)	0 (0)	0.334^*^

*Categorical data are reported as n (%). P-value: χ^2^ test with exact method for categorical variables and t-test or Mann-Whitney U test for continuous variables. P < 0.05 indicates significant statistical difference (bold values). CKD, chronic renal dysfunction; CAV, cardiac allograft vasculopathy; HBP, hypertension; HTx, heart transplantation; DM, diabetes mellitus*.

* *Fisher exact test*.

### Echocardiographic Evaluation

The results of echocardiographic evaluations, including the results of discharge and the latest follow-up results, were compared. Patients with the most recent echocardiographic evaluations accounted for 88.2 and 85.1% in the ECMO and non-ECMO groups, respectively. Some differences were observed at discharge between the groups, and these differences did not change over time during follow up; the mean SPAP of both groups notably declined in the latest echocardiographic evaluations compared to the discharge characteristics (ECMO group *P* = 0.008, non-ECMO group *P* = 0.035). The ECMO group also showed a significant improvement in tricuspid regurgitation (*P* = 0.012). However, this was not observed in the non-ECMO group. Horizontal comparisons were also performed between the groups during the same period. In terms of morphology, patients in the ECMO group had a larger RV diameter in both the discharge (*P* = 0.002) and latest (*P* = 0.004) echocardiographic evaluations.

### SPAP and RV Function

Although there was no significant difference in other quantitative indicators used to evaluate RV function, the Tei index was significantly different (*P* = 0.018) in the latest echocardiographic evaluations between the groups. The ECMO group had a higher Tei index than the non-ECMO group. Details of the echocardiographic evaluations are shown in Table 4.

**Table 4:** Latest echocardiography results.

	**ECMO (*n* = 37)**	**Non-ECMO (*n* = 97)**	***P***
EF, % (IQR)	66.0 (63.5–68.5)	63.0 (60.3–69.0)	0.693
LA diameter, mm (IQR)	38.1 (35.0–41.0)	40.0 (34.3–45.0)	0.388
RA diameter, mm (IQR)	40.0 (34.0–43.5)	35.8 (32.0–40.0)	0.094
RV diameter, mm (IQR)	23.0 (20.5–27.7)	20.8 (19.0–22.0)	**0.004**
RVOT diameter, mm (IQR)	29.0 (26.5–32.2)	26.2 (24.0–28.0)	**0.028**
FAC, % (IQR)	39.3 (31.6–49.1)	40.3 (35.6–45.4)	0.844
S', mm/s (IQR)	10.7 (9.2–11.0)	9.9 (9.1–11.3)	0.415
TAPSE, mm (IQR)	17.0 (16.0–20.9)	16.4 (15.0–17.9)	0.480
RV Tei index, (IQR)	0.46 (0.44–0.51)	0.43 (0.38–0.47)	**0.018**
SPAP, mmHg (IQR)	25.0 (25.0–30.0)	27.8 (25.0–30.0)	0.803
PAD, mm (IQR)	23.0 (22.0–24.5)	22.4 (21.0–24.0)	0.276
TR(≥moderate), *n* (%)	2 (5.4%)	7 (7.2%)	0.771

*P-value: χ^2^ test with exact method for categorical variables and t-test or Mann-Whitney U test for continuous variables*.

*P < 0.05 indicates significant statistical difference. (bold values)*

*EF, ejection fraction; LA, left atrial; RA, right atrial; RV, right ventricle; RVOT, right ventricular outflow tract; FAC, fractional area change; S', tricuspid annulus systolic velocity; TAPSE, tricuspid annular peak systolic excursion; PAD, pulmonary artery diameter; SPAP, systolic pressure of pulmonary artery; TR, tricuspid regurgitation*.

**Figure 3:**
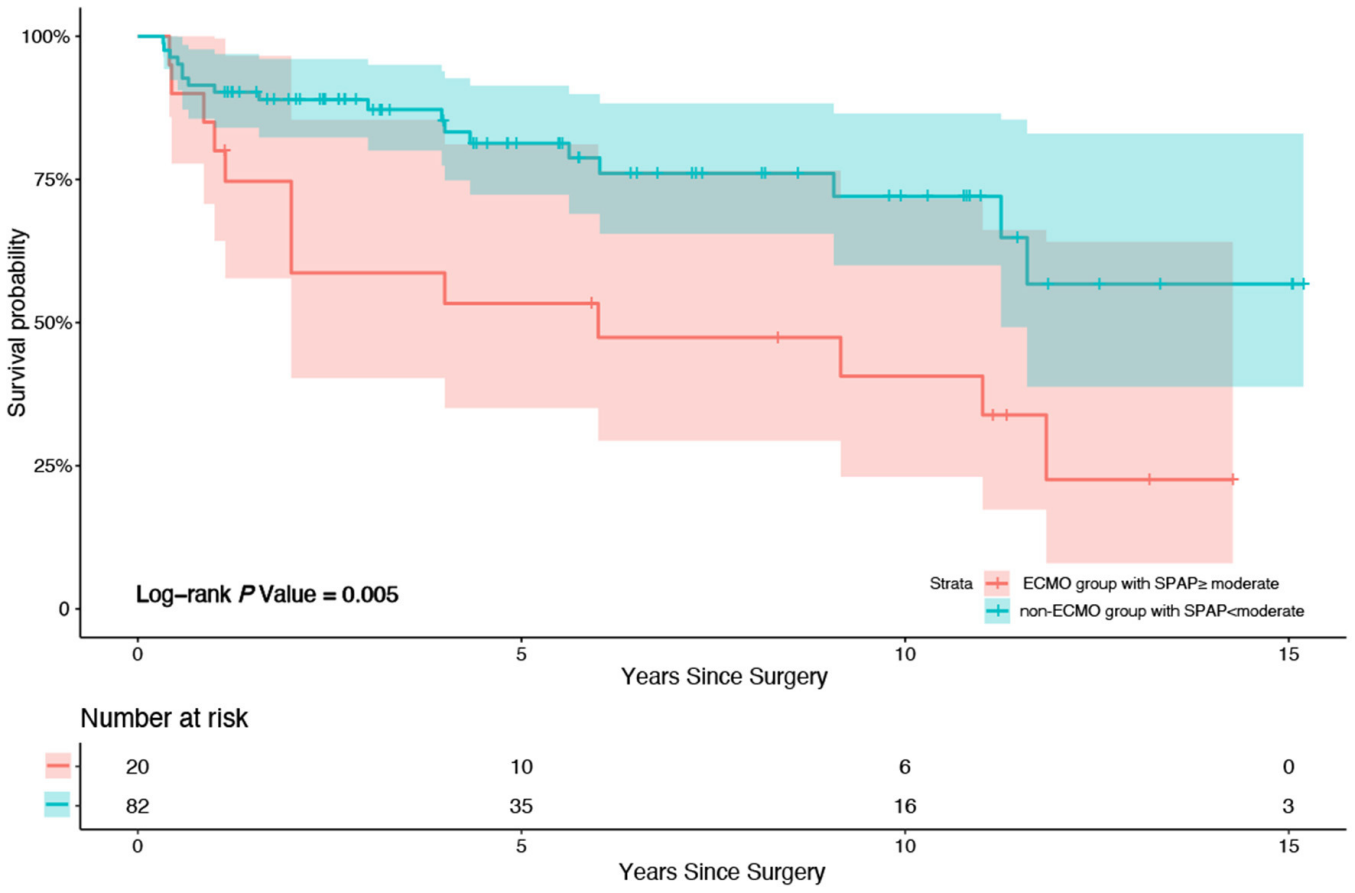
Kaplan-Meier survival analysis between patients in ECMO group with SPAP increased more than moderate (50 mmHg) after HTx before discharge from hospital and patients in non-ECMO group without SPAP increased more than moderate (50 mmHg) after HTx before discharge from hospital. Patients in the ECMO group with a more than moderate SPAP increase after HTx before discharge from hospital had higher mortality compared to patients in the non-ECMO group without a more than moderate SPAP increase after HTx before discharge from hospital (*P* = 0.005, *X*^2^ = 8.010).

Patients were divided into subgroups according to the SPAP before discharge (whether it increased more than the moderate standard of ≥50 mmHg). According to the multivariate Cox regression analysis, graft ischemic time (*P* = 0.025, HR = 1.004, 95% CI 1.000–1.007) and a more than moderate standard increase in SPAP (≥50 mmHg) before discharge (*P* = 0.002, HR = 4.316, 95% CI 1.683–11.067) were strong predictors of all-cause mortality during follow up before AIPW. A more than moderate standard increase in SPAP (≥50 mmHg) before discharge (*P* = 0.003, HR = 3.347, 95% CI 1.517–7.381) was the unique predictor of all-cause mortality after AIPW. Kaplan–Meier survival analysis was also performed; patients in the ECMO group with a more than moderate SPAP increase had higher mortality than patients in the non-ECMO group without a more than moderate SPAP increase (*P* = 0.005, *X*^2^ = 8.010) (Figure 3).

## Discussion

This study primarily showed that right-sided cardiac dysfunction in HTx recipients was a strong predictor of ECMO after HTx. Moreover, the ECMO group had significantly higher mortality than the non-ECMO group during the perioperative and follow-up periods, which may be associated with SPAP increase before discharge.

HTx is the gold standard treatment for end-stage HF (1). The most recent data from the ISHLT registry indicate that the median survival after HTx was 14.8 years (9). However, the patients in our study had a median survival of 12.0 years. The lower survival time in our study may be related to imperfect bridging before HTx in our country. As donor organs are limited, patients are often on ventricular assist device (VAD) support before receiving HTx in developed countries. Effective VAD bridging is associated with reduction in both perioperative complications and mortality in patients after HTx, especially in acute settings or under conditions of cardiac graft shortage (10, 11). Unfortunately, this alternative option has not been used in China during the last 20 years because VADs were not available until 2017 in the Chinese market. Simultaneously, owing to the unbalanced economic and educational development in our country, some patients had severe HF at hospital admission. The above reasons contributed to the lower survival time observed in our study.

LOS remains a common complication in patients early after HTx (12), and the incidence of LOS is reported to exceed 20% (13, 14). ECMO is a proven strategy for the treatment of patients with LOS (5). Our previous study showed that ECMO is acceptable for treating postcardiotomy cardiogenic shock in patients undergoing valvular surgery and was associated with good long-term outcomes in hospital survivors (6). According to the ISHLT guidelines for the management of HTx recipients, ECMO is the preferred mechanical circulatory support in patients with RV failure after HTx (15). Similarly, in this study, ECMO improved the hemodynamics of 68 of 205 (33.2%) patients. Despite growing evidence supporting ECMO use following cardiovascular surgery (4), the outcomes remain poor (16). In our study, 26 (38.2%) patients died while on ECMO support and 40 (58.8%) patients survived 30 days after ECMO implantation; this was similar to the findings of Bartko et al. where 14% of the patients died while on ECMO support and 46% died within 30 days after ECMO implantation (17).

Ischemia-reperfusion injury of the myocardium associated with graft preservation and pre-existing increased pulmonary vessel resistance are the two main causes of right-sided heart dysfunction in HTx patients postoperatively (18). In our study, CPB time, SPAP, and the pre-intervention anatomical diameters of the right side of the recipient's heart were strong predictors of ECMO. Myocardial ischemia becomes more critical with prolonged CPB time, leading to worse cardiac rebounds after weaning. As reported by Banner et al. 30-day survival decreases linearly as ischemia time increases in HTx patients (19). Moreover, reperfusion worsens the extent of tissue damage and cardiac dysfunction (20). Similarly, in this study, prolonged CPB time was a strong predictor of ECMO after HTx. The average values of anatomical diameters related to the right side of the heart were greater in the ECMO group than in the non-ECMO group; this can serve as indirect evidence of increased right-sided heart afterload. The RV diameter was directly affected by SPAP and the RVOT diameter, being an intermediary variable between the RA and RVOT diameters, which showed no significant difference in multivariate logistic regression. However, this does not imply that there was no difference in the RV diameter between the groups. The morphological changes and increased SPAP suggested that the right-sided heart function of patients in the ECMO group was already impaired preoperatively. To ameliorate the right-sided heart dysfunction caused by myocardial ischemia and increased pulmonary vascular resistance, ECMO was performed for critical patients at our center.

The pathophysiological and prognostic significance of the right side of the heart was previously underestimated but has been recognized to have crucial prognostic significance (21). Bartko et al. suggested that the metrics of RV function were the strongest predictors of outcomes in patients who underwent cardiac surgery (17), which was in line with the results of our study. The ECMO group still showed significantly higher mortality than the non-ECMO group during follow up, even after adjustment. After a median follow-up period of 53 months, 35.0% (14 patients) and 25.0% (31 patients) of patients died in the ECMO and non-ECMO groups, respectively. This may suggest that patients requiring ECMO after HTx have a limited prognosis if right-sided heart function is impaired preoperatively.

In the latest echocardiographic evaluation in our study, the ECMO group had a larger mean RV diameter and RV outflow diameter than the non-ECMO group. Although there was no significant difference in other function parameters, such as FAC, S', and TAPSE, the Tei index was significantly associated with RV function. The Tei index is defined as the sum of the isovolumic contraction and relaxation times divided by the ejection time; it incorporates both systolic and diastolic time intervals to express global systolic and diastolic ventricular function (22, 23). Therefore, the Tei index avoids the irregular anatomical shape of the right side of the heart and the tracing error caused by the endocardial myocardium trabecula in echocardiography when measuring FAC, S', and TAPSE. Previous studies have shown that the RV Tei index is significantly related to SPAP (24) and is elevated owing to increased pulmonary vascular resistance and decreased myocardial contraction (25). The ECMO group had a higher RV Tei index than the non-ECMO group, suggesting that these patients had increased pulmonary vascular resistance and decreased myocardial contraction during follow up. There was no significant difference in pulmonary pressure between the groups, which is probably related to the measurement method. In our center, we routinely use the tricuspid regurgitation method to non-invasively measure pulmonary pressure because right heart catheterization is an invasive procedure with associated risks that significantly limit its utility for routine monitoring of pulmonary hypertension. Indeed, SPAP is a standard assessment in contemporary echocardiography and can be measured in approximately two thirds of all echocardiograms. A good correlation has been found between right heart catheterization and echocardiography (26).

The progression of pulmonary vessel disease also plays an important role in the development of cardiovascular diseases. In a study by Crawford et al. (27), SPAP was associated with significant increase after HTx, which was in line with our findings. SPAP was a strong predictor of all-cause mortality during follow up in the multivariate Cox regression analysis in our study. In the subgroup analysis performed based on SPAP, the prognosis of patients in the ECMO group with more than moderate SPAP increase was much lower than that of the patients in the non-ECMO group without a more than moderate increase (*P* = 0.005, *X*^2^ = 8.010). This suggests that patients requiring ECMO after HTx have a limited prognosis if the pulmonary vessels are impaired preoperatively. Han et al. (28) reported that cardiovascular involvement caused by pulmonary vessel disease is particularly relevant and is associated with impaired health status and worse mortality. Previous studies have found that in patients with rheumatic heart disease, even when rheumatism had been controlled and the implicated valves replaced, the damage to the pulmonary capillary vessels remained irreversible (29), showing the same trend as that observed in our study. Among patients with end-stage HF, LV systolic and diastolic function decreased, which led to increased filling pressures in the left heart. This initiates a series of adverse pathological and functional changes in the pulmonary vasculature and eventually in the right side of the heart. We consider that the most important and direct cause of right-sided heart dysfunction is the pre-HTx changes in the pulmonary vessels, which exist even after a healthy heart is replaced. Elevated pressure of the recipient's pulmonary artery leads to insufficiency of the right-sided heart, which leads to the application of ECMO early after HTx, also leading to higher mortality during long-term follow up. However, the changes in pulmonary vessels require further confirmation by pathology studies.

### Study Strengths

To our knowledge, our study is the first retrospective study to investigate the impact of ECMO on right-sided heart performance in terms of both morphology and function after HTx. There were no significant differences in the baseline characteristics between the two groups except for some echocardiographic characteristics that cannot be ruled out. All survival analyses and right-sided heart evaluations were based on real-world data, which could be a reliable reference for clinicians to make treatment decisions for HTx patients, especially for those requiring ECMO.

### Study Limitations

Our study was mainly limited by the small sample size, which made it difficult to determine reliable relationships among observed events through statistical analysis. For instance, the ECMO group had a high mortality rate, as such echocardiographic data were available for only a relatively small subset of patients. Additionally, a biopsy is difficult to perform regularly after HTx in China because of problems with cooperation. If biopsy was performed, the changes in the right side of the heart and pulmonary vessels would have been better evaluated. Although normal echocardiographic characteristics were measured at hospital admission during follow-up, there was no tracking of continuous changes in the assessment of right-sided heart function (RV tei index, S', and FAC). Especially there were some deficiencies in the echocardiographic data in agonal stage, we cannot determine whether the main cause of death was pulmonary hypertension or right heart failure. Finally, although differences in the measured covariables were minimized after propensity score weighting, unmeasured and unknown covariables were probably present. Therefore, further studies are required to confirm our findings.

## Conclusions

Recipient right-sided heart characteristics were strong predictors of ECMO after HTx. ECMO patients had high mortality in the perioperative and follow-up periods, and the changes in RV function in ECMO patients may be associated with pulmonary vessel injury before and after HTx. Further studies are needed to assess the impact of ECMO on right-sided heart function.

## Data Availability Statement

The raw data supporting the conclusions of this article will be made available by the authors, without undue reservation.

## Ethics Statement

Ethics approval for this study was obtained from the Research Ethics Committee of Beijing Anzhen Hospital, Capital Medical University, Beijing, China. IRB No. 2021096X Approve date: Jul. 6, 2020. The patients/participants provided their written informed consent to participate in this study. Written informed consent was obtained from the individual(s) for the publication of any potentially identifiable images or data included in this article.

## Author Contributions

All authors listed have made a substantial, direct, and intellectual contribution to the work and approved it for publication.

## Funding

This work was supported by the National Natural Science Foundation of China (82170311, 81770320, and 81570291).

## Conflict of Interest

The authors declare that the research was conducted in the absence of any commercial or financial relationships that could be construed as a potential conflict of interest.

## Publisher's Note

All claims expressed in this article are solely those of the authors and do not necessarily represent those of their affiliated organizations, or those of the publisher, the editors and the reviewers. Any product that may be evaluated in this article, or claim that may be made by its manufacturer, is not guaranteed or endorsed by the publisher.
